# Scorpion neurotoxin AaIT-expressing *Beauveria bassiana* enhances the virulence against *Aedes albopictus* mosquitoes

**DOI:** 10.1186/s13568-017-0422-1

**Published:** 2017-06-09

**Authors:** Sheng-Qun Deng, Qun-Di Cai, Ming-Zhi Deng, Qiang Huang, Hong-Juan Peng

**Affiliations:** Department of Pathogen Biology, Guangdong Provincial Key Laboratory of Tropical Disease Research, and Key Laboratory of Prevention and Control for Emerging Infectious Diseases of Guangdong Higher Institutes, School of Public Health, Southern Medical University, Guangzhou, 510515 Guangdong Province China

**Keywords:** *Beauveria bassiana*, *Aedes albopictus*, Scorpion neurotoxin, Virulence

## Abstract

To improve the insecticidal efficacy of this entomopathogen *Beauveria bassiana*, the fungus was genetically modified to express an insect-specific scorpion neurotoxin AaIT. The virulence of the recombinant *B. bassiana* strain (*Bb*-AaIT) against *Aedes albopictus* adults (which occurs via penetration through the cuticle during spore germination or by conidia ingestion), and the larvae (by conidia ingestion) was measured with bioassays. The median lethal concentration (LC_50_) of *Bb*-AaIT against *A. albopictus* larvae was 313.3-fold lower on day 4 and 11.3-fold lower on day 10 than that of the wild type (WT). Through conidia feeding or body contact, *Bb*-AaIT killed 50% of adult female mosquitoes at 3.9- or 1.9-fold reduced concentrations on day 4 and at 2.1- or 2.4-fold reduced concentrations on day 10. Compared with the results for the WT, the median lethal time (LT_50_) of *Bb*-AaIT was reduced by 28.6% at 1 × 10^7^ conidia ml^−1^ and 34.3% at 1 × 10^6^ conidia ml^−1^ in the larvae bioassay by conidia ingestion, while it decreased 32.3% at 1 × 10^7^ conidia ml^−1^ by conidia ingestion and 24.2% at 1 × 10^8^ conidia ml^−1^ by penetrating through the cuticle in the adult bioassay. All the differences were significant. Our findings indicated that *Bb*-AaIT had higher virulence and faster action than the WT in killing the larval and adult mosquitoes, and therefore, it is valuable for development as a commercial mosquito pesticide.

**Electronic supplementary material:**

The online version of this article (doi:10.1186/s13568-017-0422-1) contains supplementary material, which is available to authorized users.

## Introduction

Mosquito-borne diseases create a significant burden every year. For example, malaria caused 438,000 deaths in 2015, and the dengue incidence has risen 30-fold in the past 30 years around the world (WHO 2016). The Asian tiger mosquito *Aedes albopictus* transmits many viral infections, including the yellow fever, dengue and Chikungunya (Hochedez et al. 2006), as well as several filarial nematodes such as *Dirofilaria immitis* (Cancrini et al. 2003). *Aedes albopictus* is capable of hosting the Zika virus and is considered a potential vector for Zika transmission among humans (Paupy et al. 2009; Wong et al. 2013). Because more than half of the world’s population lives in areas where this mosquito species is present, sustained mosquito control efforts are important to prevent outbreaks from these diseases (Kamareddine et al. 2013).

Chemical insecticides have been used intensively as the primary strategy for controlling mosquito populations. However, this strong dependence on insecticides for mosquito control around the world and the use of these chemicals in agriculture have led to environmental pollution and toxic hazards to humans and other non-target organisms (Al-Sarar 2010). The physiological resistance of important mosquito vectors has been widely reported in recent years (Agra-Neto et al. 2014; Bilal et al. 2012; Labbe et al. 2007; Lima et al. 2011; Stenhouse et al. 2013; Zou et al. 2006).

Entomopathogenic fungi, such as *Beauveria bassiana* and *Metarhizium anisopliae*, have tremendous potential for use as pest biological control agents, particularly as alternatives to chemical pesticides (Lacey et al. 2001; Lomer et al. 2001). *B. bassiana* is an environmentally friendly fungus (Roberts and St Leger 2004; Thomas and Read 2007; Wang et al. 2004), and it is widely distributed and has a broad host range towards diverse insect species in nature (Reynolds and Samuels 1996). Many *B. bassiana* strains have been selected for vector and crop pest control, including house and fruit flies (Dimbi et al. 2003; Lecuona et al. 2005), mosquitoes (Bukhari et al. 2011; Clark et al. 1968; Garcia-Munguia et al. 2011), ticks (Kirkland et al. 2004), locusts, grasshoppers, and termites (Kassa et al. 2004; Mburu et al. 2009). However, the slow killing speed associated with these mycoinsecticides is a major deterrent to their commercial use and large-scale application, and thus they are unable to compete with faster-acting and cheaper chemical insecticides (Amsellem et al. 2002; St Leger et al. 1996).

AaIT is a single-chain neurotoxic polypeptide that is derived from the venom of the buthid scorpion *Androctonus australis*, and its strict selective toxicity for insects has long been documented (Dee et al. 1990; Zlotkin et al. 2000). It has been reported that the integration of AaIT into *M. anisopliae* for specific expression in the insect hemolymph after cuticle penetration resulted in 22-, 9-, and 16-fold increases in fungal toxicity to the tobacco hornworm *Manduca sexta*, the yellow fever mosquito *Aedes aegypti*, and the coffee berry borer *Hypothenemus hampei*, respectively (Pava-Ripoll et al. 2008; Wang and St Leger 2007). Similarly, *B. bassiana* expressing AaIT presented a 15-fold increase in insecticidal activity against Masson’s pine caterpillar *Dendrolimus punctatus*, and its median lethal times (LT_50_) against the larvae of *D. punctatus* and *Galleria mellonella* were reduced by 24.4 and 40%, respectively (Lu et al. 2008). In this study, we successfully improved the pathogenicity of *B. bassiana* against *A. albopictus* by engineering it to express the scorpion neurotoxin AaIT. The virulence of the recombinant fungi against adult *A. albopictus* (through cuticle penetration or conidia ingestion) and larvae (through conidia ingestion) was significantly improved.

## Materials and methods

### Mosquitoes

The Foshan (Guangdong, China) strain of *A. albopictus* originated in Foshan, Guangdong Province, PRC, and it was established in our laboratory in 1981. All mosquitoes, including larval and adult mosquitoes, were maintained in humidified incubators at 25  ±  1 °C on a 12-h light: dark photocycle.

### Microbial strains and media

The *B. bassiana* GIM3.428 strain (wild type, WT) was purchased from Guangdong Microbiology Culture Center and maintained on Czapek’s agar (CDA) (w/v: 3.0% sucrose, 0.3% NaNO_3_, 0.1% K_2_HPO_4_, 0.05% KCl, 0.05% MgSO_4_·7H_2_O, 0.001% FeSO_4_ and 2% agar) at 4 °C for preservation and at 25 °C for colony growth. The WT *B. bassiana* that was used as the parental strain in this study was sequentially cultured in Sabouraud dextrose broth (SDB) (w/v: 4% glucose, 1% peptone and 1% yeast extract) and glucose-mineral (GM) medium (w/v: 4% glucose, 0.4% NH_4_NO_3_, 0.3% KH_2_PO_4_ and 0.3% MgSO_4_) for blastospore production. *Escherichia coli* Trans-T1 cells (Tansgen, Beijing, CHA), which are used for DNA manipulation, were cultured in Luria–Bertani medium containing 100 µg/ml ampicillin or 50 µg/ml kanamycin.

### Gene synthesis and vector construction

The coding sequence of AaIT was synthesized by using *B. bassiana*-preferred codons and the Mcl1 signal peptide sequence (SP) was incorporated at the 5′ end of it for secretion expression (Genebank accession number: KY914486, Additional file 1: Figure S1a) (Wang and St Leger 2007). The synthesized gene was bracketed with *EcoR*I and *Xho*I sites and cloned into pBARGPE1, forming the plasmid pBARGPE1-AaIT (Additional file 1: Figure S1b). This plasmid retains a strong gpdA promoter to drive the insert’s gene expression and Bar resistance coding sequence to express the selectable marker that would provide resistance to phosphinothricin (PPT). The recombinant plasmid pBARGPE1-AaIT was transformed into *E. coli* Trans-T1 for propagation, followed by isolation with Plasmid Mini Kit I (Omega, Norcross, GA). After it was linearized with *Sca*I (Thermo Fisher Scientific, Waltham, MA), the plasmid was purified using a Cycle-Pure Kit (Omega, Norcross, GA) and dissolved in dd-H_2_O at a concentration of 100 ng/µl for blastospore electro-transformation.

### Blastospore preparation

*Beauveria bassiana* conidia formation was triggered by culturing the fungi in SDB with 120 rpm shaking at 25 °C for 48 h, and then 5 ml of this fungal culture was transferred to 50 ml of GM medium for 12 h with shaking under the same conditions. The resulting cultures were filtered through layers of sterile cotton to get rid of the hyphae, and the blastospores were harvested by a 10 min centrifugation cycle at 5000×*g* and 4 °C, followed by two washes with dd-H_2_O. The harvested blastospores were suspended with 0.5 ml of 1 M sorbitol and counted with a hemocytometer. Each suspension was mixed with an appropriate amount of 80% sterile glycerol to dilute the blastospore concentration to 1 × 10^8^/ml, and the samples were ultimately stored at −80 °C for sequential use in electro-transformation.

### Electroporation

The stored blastospores were precipitated after centrifugation at 5000×*g* and 4 °C for 5 min, and they were suspended in the mixture containing 10 µl of linearized pBARGPE1-AaIT and 90 µl of ice-cold 1 M sorbitol. The suspension was then transferred into a 0.2 mm prechilled cuvette and pulsed using an electroporator (Bio-Rad, CA, USA) at a field strength of 2 kV cm^−1^ for 5 ms (milliseconds). The pulsed suspension was transferred into a new Eppendorf tube. The residue in the cuvette was washed twice with 150 µl of 1 M ice-cold sorbitol and 300 µl of washing solution, and the washing solution was also added to the Eppendorf tube. The final suspension was incubated for 2 h at 25 °C and then 150 µl aliquots were smeared on CDA plates containing 150 µg/ml PPT.

### Isolation of recombinants

Each transformation product was grown on CDA plates containing 150 µg/ml PPT at 25 °C. The putative recombinants were picked out and subcultured for three generations on CDA with 150 µg/ml PPT. The genomic DNA of every generation was extracted from the 4-day-old colonies according to a documented method (Raeder and Rroda 1985) and used as PCR templates to detect the *AaIT* gene presence in the recombinants with SP-AaIT-F and SP-AaIT-R primers (5′-CCCAAGCTTATGCGTGAACTTTCTTCGGT-3′ and 5′-CCCTCGAGTTAGTTGATGATGGTGGTAT-3′, respectively). To verify the mitotic stability of their PPT resistance, the recombinants were subcultured for three generations on CDA without PPT at 25 °C, and finally, they were subcultured on CDA with 400 µg/ml PPT at 25 °C. Genomic DNAs were extracted from the 4-day-old colonies from the last subculture and then analyzed by PCR. A stable recombinant strain named *Bb*-AaIT was selected for the subsequent experiments.

### Identification of AaIT expression in *Bb*-AaIT

The WT and *Bb*-AaIT strains were grown in CDA for 4 days. Dead female adult mosquitoes that were infected by ingesting WT or *Bb*-AaIT conidia were maintained at 25 °C at saturated humidity for 4 days. To verify the transcription of *AaIT* gene in *Bb*-AaIT, the total RNAs were extracted from the CDA culture supernatant or infected mosquitoes using an RNeasy mini plant kit (Qiagen, Duesseldorf, GER), and they were subjected to reverse transcription with random primers and then PCR with the primers RT-AaIT-F and RT-AaIT-R (5′-CTTTCTTCGGTTCTCGCCCT-3′ and 5′-CCTTATCGGCGTAGTGGACC-3′, respectively).

A polyclonal antibody against AaIT that was expressed by *E. coli* BL21 (DE3) was derived from rabbits and used to detect the expression of AaIT in the *Bb*-AaIT or WT grown in CDA or in the infected mosquitoes by western blotting. To verify the translation of *AaIT* gene in *Bb*-AaIT, the fungi that were precipitated from the CDA culture supernatant and the infected mosquitoes were ground under liquid nitrogen in mortars for 5 min. Then, 1 ml of cell lysis solution (Beyotime, Shanghai, CHA) was added to the mortars, and the samples were further ground on ice for another 5 min. The ground-up mix was transferred to an Eppendorf tube, and after centrifugation at 12,000 rpm for 5 min, the pellets were reconstituted in sterile ddH_2_O. The concentrations of the total protein extracts were measured by Nanodrop (Thermo Fisher Scientific, Waltham, MA). Fifty microgram of total protein extracts were loaded for SDS-PAGE, and then western blotting was performed.

### Bioassays

The conidia of the *Bb*-AaIT and WT strains were harvested from the CDA plates with sterile cotton swabs, resuspended in ddH_2_O, and counted with a hemocytometer. Three bioassays were conducted to compare the virulence of the *Bb*-AaIT and WT strains to the second-instar larvae of *Aedes albopictus* infection through conidia ingestion (assay 1), 3-day-old (3 days after emergence) female adult mosquitoes infection through conidia ingestion (assay 2) or through cuticle contact (assay 3). In assay 1, conidia suspensions of 1 × 10^5^ conidia/ml (low), 1 × 10^6^ conidia/ml (middle) and 1 × 10^7^ conidia/ml (high) of *Bb*-AaIT and WT were added to the cups containing 20 s-instar *A. albopictus* larvae in 30 ml of double distilled water, respectively. Each treatment was performed in three replicates, and the mortality levels of the larvae were recorded every 24 h for 10 days. In assays 2 and 3, the 3-day-old adult female mosquitoes were anesthetized with a multiunit CO_2_ anesthetizing system (Vessey et al. 2008). In assay 2, 30 anesthetized female mosquitoes in each group were transferred to the plastic container immediately and fed with 10% glucose containing *Bb*-AaIT and WT conidia. The conidia had different concentrations of 1 × 10^6^ conidia/ml (low), 1 × 10^7^ conidia/ml (medium) and 1 × 10^8^ conidia/ml (high), respectively, and the control group was fed pure 10% glucose. The tests were conducted in the laboratory at 25 °C. In assay 3, 30 anesthetized female mosquitoes in each group were placed on the 50 cm^2^ filter paper that had absorbed 3 ml of conidial suspension at 1 × 10^5^ conidia/ml (low), 1 × 10^6^ conidia/ml (middle) and 1 × 10^7^ conidia/ml (high) or 0.02% Tween 80 (control) for 10 s. The mosquitoes were transferred separately to different plastic containers. The contaminated mosquitoes or control mosquitoes were maintained on a 10% glucose diet in plastic containers at 25 °C. Each treatment was performed in three replicates and the mortality levels of the adult female mosquitoes were recorded every 12 h during assays 2 and 3.

A Kaplan–Meier survival analysis and a log-rank test were used to compare the differences between these two strains at three concentrations and to compare the difference between these two strains at each given concentration. A time–concentration–mortality (TCM) model analysis (Feng et al. 1998; Nowierski et al. 1996) was used to calculate the median lethal concentration (LC_50_) and the median lethal time (LT_50_) in the treated mosquitoes. All the tests were conducted with IBM SPSS (ver. 20.0) and DPS software (ver. 7.05) (Tang and Feng 2007).

## Results

### Detection of AaIT gene presence and the mitotic stability of the *Bb*-AaIT strain

Transformation of the competent blastopores of WT with linearized plasmid pBARGPE1-AaIT produced one transgenic colony on the CDA plate containing 150 µg/ml PPT. The tansformant was able to grow on CDA plate containing 150 µg/ml PPT for three generations. After three rounds of subculturing on PPT-free CDA plates, the tansformant be capable of growing on the CDA plate containing 400 µg/ml PPT. The genomic DNA of the colonies grown on the plate during each generation were extracted and used as PCR templates to detect the presence of the *AaIT* gene in *Bb*-AaIT. The expected PCR fragments from all the *Bb*-AaIT samples appeared on the agarose gel, but not the WT (Additional file 1: Figure S2), which confirmed the consistent heredity of the *AaIT* gene in the *Bb*-AaIT genome.

### Detection of AaIT expression in the *Bb*-AaIT strain

**Fig. 1:**
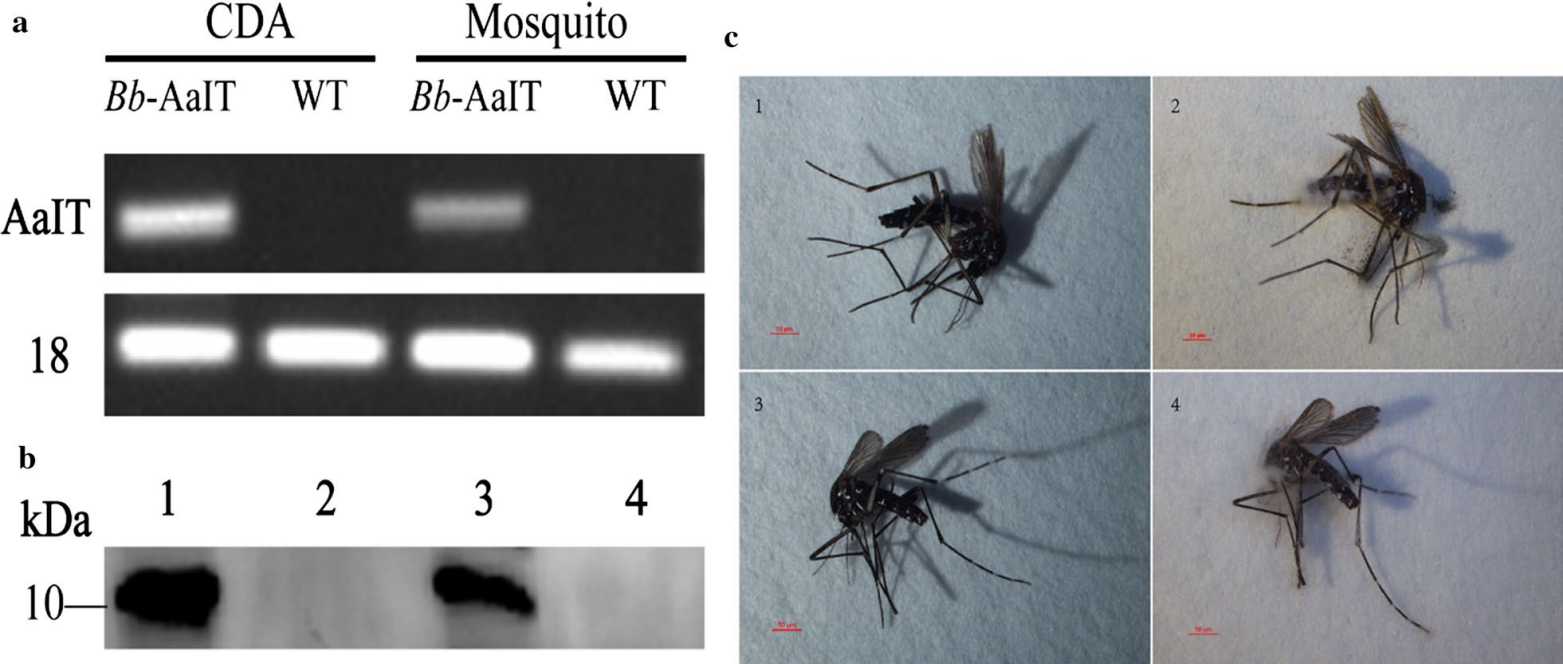
Evidence for AaIT expression in the *Bb*-AaIT strain. **a** RT-PCR detection of *AaIT* gene transcription in *Bb*-AaIT collected from the infected dead mosquitoes and the CDA with positive results, and in the WT control with negative results. The 18S rRNA was detected for the loading control. **b** Western blot detection of AaIT expression in *Bb*-AaIT and the WT with a polyclonal antibody against AaIT from different samples. *Lane 1*, culture supernatant from *Bb*-AaIT grown in CDA for 4 days with a positive result; *Lane 2*, culture supernatant from WT grown in CDA for 4 days with a negative result; and *Lane 3*, the dead female mosquitoes infected by *Bb*-AaIT and incubated at 25 °C for another 4 days. *Lane 4*, the dead female mosquitoes infected by the WT and incubated at 25 °C for another 4 days. **c** The dead female mosquitoes were infected by *Bb*-AaIT or WT through conidial ingestion. Fungal outgrowths were not observed in the uncultured dead mosquitoes (*1* and *3*), and typical fungal outgrowths were observed on the dead mosquitoes that were infected by *Bb*-AaIT (*2*) and the WT (*4*) after 4 days of incubation at 25 °C and saturated humidity. *Bars*, 50 µm

The WT and *Bb*-AaIT strains were grown in CDA for 4 days at 25 °C and precipitated with centrifugation, and they were kept for analysis. The fungal outgrowths were those of a typical *B. bassiana*, and they appeared on the dead female mosquitoes that were infected through conidial ingestion after being maintained at 25 °C at saturated humidity for 4 days (Fig. 1c). The total RNAs and proteins of the WT and *Bb*-AaIT from the culture supernatant or infected mosquitoes were extracted and analyzed by RT-PCR (Fig. 1a) or western blotting (Fig. 1b). This result indicated that the recombinant strain expressed the AaIT toxin consistently, but not the WT strain.

### Bioassay results for the *Bb*-AaIT and WT against larval or adult mosquitoes

**Fig. 2:**
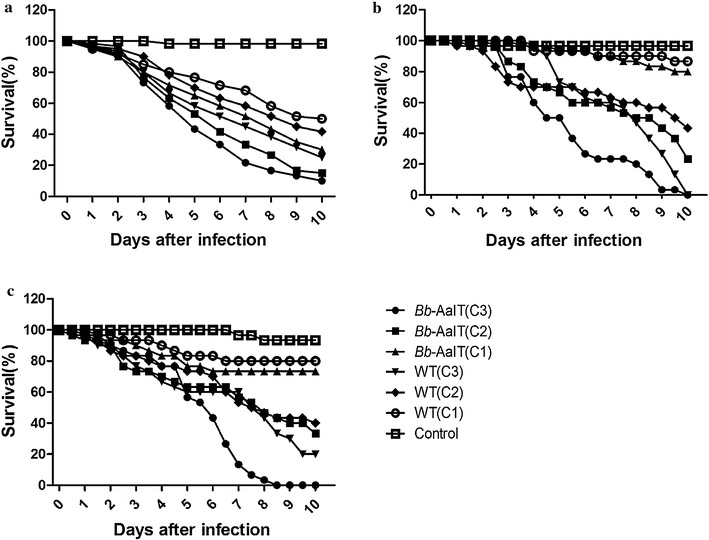
Survival curves of larval and adult mosquitoes for different *Bb*-AaIT and WT treatments. **a** The survival curve of *Aedes albopictus* larvae when treated with (C1) 1 × 10^5^, (C2) 1 × 10^6^ and (C3) 1 × 10^7^ conidia ml^−1^ suspensions of *Bb*-AaIT and the WT. **b** The survival curve of *Aedes albopictus* female adults that were infected by conidia ingestion with (C1) 1 × 10^5^, (C2) 1 × 10^6^ and (C3) 1 × 10^7^ conidia ml^−1^ suspensions of *Bb*-AaIT and WT. **c** The survival curve of *Aedes albopictus* female adults that were infected through cuticle contact with 3 ml of (C1) 1 × 10^6^, (C2) 1 × 10^7^ and (C3) 1 × 10^8^ conidia ml^−1^ suspensions containing *Bb*-AaIT and WT that were sprayed on 50 cm^2^ filter paper

Bioassays for the pathogenicity of the *Bb*-AaIT and WT against the 2-instar larvae and the female adults of *Aedes albopictu*s mosquitoes (through cuticle penetration or conidia ingestion) were conducted at low, middle, and high conidial concentrations. Significant differences were found among the different *Bb*-AaIT or WT treatment concentrations (Table 1). The mortalities generally increased with the increased conidial concentration and the post-treatment time for both *Bb*-AaIT and WT (Fig. 2).

**Table 1:** Results of the log-rank test for the different concentrations of *Bb*-AaIT or the WT against *Aedes albopictus* (larval or female adult mosquitoes) in assays 1–3

Assays	Fungal strains	χ^2^	Df	*P*
Assay 1	*Bb*-AaIT	10.155	2	0.006
	WT	8.402	2	0.015
Assay 2	*Bb*-AaIT	50.706	2	<0.001
	WT	39.299	2	<0.001
Assay 3	*Bb*-AaIT	30.904	2	<0.001
	WT	18.077	2	<0.001

*P* < 0.05 means that the difference is significant

In assay 1, significant differences were found between the mortalities of *Bb*-AaIT and the WT-treated larvae at each given concentration (Table 1). That is, the larvae that were treated with *Bb*-AaIT tended to die faster than those treated with the WT (Fig. 2a), and the difference was significant (Table 2). In assays 2 and 3, *Bb*-AaIT-treated mosquitoes tended to die faster than those treated with the WT at the high concentration and the difference was significant; however, no significant difference was found between the *Bb*-AaIT and WT-treated adult mosquitoes at the low and middle concentrations (Fig. 2b, c; Table 2).

**Table 2:** Results of the log-rank test on the different fungal strains *Bb*-AaIT and WT against *Aedes albopictus* (larval or female adult mosquitoes) at each given concentration in assays 1–3

Assays	Concentrations (conidial ml^−1^)	χ^2^	df	*P*
Assay 1	1 × 10^7^	5.999	1	0.014
	1 × 10^6^	10.898	1	0.001
	1 × 10^5^	4.677	1	0.031
Assay 2	1 × 10^7^	9.574	1	0.002
	1 × 10^6^	1.365	1	0.243
	1 × 10^5^	0.451	1	0.502
Assay 3	1 × 10^8^	10.612	1	0.001
	1 × 10^7^	0.160	1	0.689
	1 × 10^6^	0.399	1	0.528

*P* < 0.05 means that the difference is significant

**Fig. 3:**
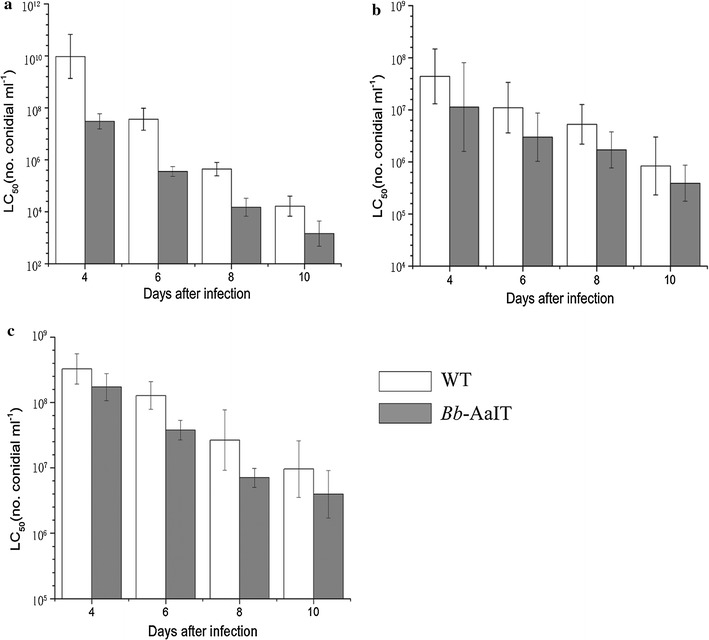
LC_50_ of WT and *Bb*-AaIT strains against larval or adult mosquitoes. *Error bars*: ± SE. Three bioassays were conducted to compare the virulence of the *Bb*-AaIT and WT strains as follows: **a** assay 1, the second-instar larvae of *Aedes albopictus* infection through conidia ingestion. (**b**, **c**) 3-day-old (3 days after emergence) female adult mosquito infection through cuticle contact (**b**, assay 2) or through conidia ingestion (**c**, assay 3)

The median lethal concentrations were calculated by using the TCM model (Feng et al. 1998; Qin et al. 2010). No significant heterogeneity was detected for each of the fitted TCM relationships (*P* > 0.05 in Hosmer–Lemeshow tests for the goodness of fit). As a result of the modeling analyses, the LC_50_s of *Bb*-AaIT and the WT against the larvae were estimated to be 3.04 × 10^7^ and 9.52 × 10^9^ conidia ml^−1^ on day 4 and then they decreased to 1.47 × 10^3^ and 1.65 × 10^4^ conidia ml^−1^ on day 10 (Fig. 3a). For assay 2, the LC_50_ estimated for *Bb*-AaIT and the WT against female mosquitoes that were infected through the cuticle dropped from 1.13 × 10^7^ and 4.39 × 10^7^ conidia ml^−1^ on day 4 to 3.91 × 10^5^ and 8.38 × 10^5^ conidia ml^−1^ on day 10 (Fig. 3b), respectively. For assay 3, the LC_50_ estimated for *Bb*-AaIT and the WT against female mosquitoes infected through conidial ingestion were 1.71 × 10^8^ and 3.26 × 10^8^ conidia ml^−1^ on day 4, and then decreased to 3.96 × 10^6^ and 9.56 × 10^6^ conidia ml^−1^ on day 10 (Fig. 3c), respectively. The LC_50_s of the *Bb*-AaIT conidia decreased with the treatment time in assays 1, 2 and 3 from 313.3-, 3.9- and 1.9-fold on day 4 to 11.3-, 2.1- and 2.4-fold on day 10, respectively, when compared with those of the WT conidia.

The median lethal times were estimated by using the TCM model. The LT_50_s of *Bb*-AaIT and WT could not be calculated at the low concentration because no more than half the quantity of lethal larvae or adult mosquitoes could be observed at the final time. The LT_50_ trends for *Bb*-AaIT were lower than those of the WT at the middle and high concentrations in the three assays. For instance, the LT_50_s of the treated larvae were 7.7 and 6.7 days for the WT and 5.5 and 4.4 days for *Bb*-AaIT at the middle and high concentrations, respectively (Table 3). The LT_50_s for *Bb*-AaIT were reduced by 28.6 and 34.3% at these two concentrations in assay 1 when compared with that of WT. The LT_50_s of the treated adult female mosquitoes at the middle and high concentrations were 9.9 and 6.2 days for the WT and 8.8 and 4.2 days for *Bb*-AaIT, respectively, in assay 2, 9.8 and 6.2 days for WT and 7.5 and 4.7 days for *Bb*-AaIT, in assay 3 (Table 3). The LT_50_ differences between the two strains reached 11.1, and 32.3% in assay 2 and 23.5 and 24.2% in assay 3 at middle and high concentrations, respectively, but no significant difference was found for the middle concentration from both assays. This difference indicated the substantial impact of the expressed AaIT toxin on the survival of the tested larvae and adult mosquitoes.

**Table 3:** The median lethal times (LT_50_s) of the fungal strains *Bb*-AaIT and WT against *Aedes albopictus* (larval or female adult mosquitoes) at middle and high concentrations in assays 1–3

Concentrations (conidial ml^−1^)	Assay 1 (LT_50_, days)		Assay 2 (LT_50_, days)		Assay 3 (LT_50_, days)	
	1 × 10^7^ (High)	1 × 10^6^ (Middle)	1 × 10^7^ (High)	1 × 10^6^ (Middle)*	1 × 10^8^ (High)	1 × 10^7^ (Middle)*
*Bb*-AaIT	4.4	5.5	4.2	8.8	4.7	7.5
WT	6.7	7.7	6.2	9.9	6.2	9.8

* The difference of LT_50_ for two strains is not significant

## Discussion

The insect-specific toxin AaIT is toxic to larval and adult mosquitoes (Dee et al. 1990; Higgs et al. 1995), and it specifically affects the voltage-gated sodium channels (VGSCs) in insects (Gurevitz et al. 2007). Although VGSCs in different taxa show high similarities, AaIT exhibits high specific sensitivity to insects and distinguishes between insects and mammals, which makes it particularly well-suited for the development of safe bioinsecticides (Zlotkin et al. 2000). AaIT induces the fast excitatory paralysis caused by a presynaptic effect, or the induction of a repetitive firing in the terminal branches of the insect’s motor nerves, which results in the massive and uncoordinated stimulation of the respective skeletal muscles (Gurevitz et al. 2007; Zlotkin et al. 2000). The adult mosquitoes that were infected by *M. anisopliae* expressing AaIT demonstrated pre-lethal effects including spasmodic leg and wing movements several hours before death, and they invariably died with extended wings, which is indicative of the muscle contraction resulting from AaIT (Wang and St Leger 2007).

In this study, the amino acid sequence of the AaIT protein was obtained from the Swiss-Prot database. In the optimized sequence, we used the preferred codons of *B. bassiana* to encode all of the given amino acids, which would improve the gene expression level (Elena et al. 2014). The coding sequence of AaIT was synthesized and cloned into pBARGPE1 (Additional file 1: Figure S1b). The strong gpdA promoter can drive the exogenous gene expression efficiently, which had been extensively and successfully applied for the transgenic gene expression in *M. anisopliae* and *B. bassiana* (Lu et al. 2008; St et al. 1996). Furthermore, the *AaIT* gene was genetically induced into the genome of the wild type *B. bassiana* to enhance its virulence to mosquitoes, and the mitotically stable transformant *Bb*-AaIT could successfully express this exogenous toxin. The virulence of *Bb*-AaIT and the wild type to larval (with infection through conidia ingestion) or adult mosquitoes (infection through cuticle or conidia ingestion) were compared at low, middle and high concentrations by survival analysis. For both *Bb*-AaIT and the WT, the virulence was increased with the increase in the concentration, and the difference was significant. *Bb*-AaIT-treated larvae tended to die faster than those treated with the WT at each given concentration; and *Bb*-AaIT-treated adult mosquitoes only died faster than those treated with the WT at the high concentration. The difference was significant.

The TCM modeling can reflect the integrity and objectivity of the bioassay data, which accounts for the effects of the conidial concentration and post-treatment time as well as the interaction of both variables (Feng et al. 1998; Nowierski et al. 1996). The robust TCM modeling analysis clearly differentiated the LC_50_ and LT_50_ trends in *Bb*-AaIT and the WT during the three bioassays on larvae and adult mosquitoes. In terms of the LC_50_, *Bb*-AaIT was substantially improved in terms of virulence compared to the WT strain. With the same treatments, the LT_50_s of the *Bb*-AaIT strain at the middle and high conidial concentrations were shortened by 11.1–34.3%, respectively, compared to the WT strain. Apparently, the higher level of virulence and the faster action of *Bb*-AaIT were attributed to the insecticidal activity of the AaIT that was expressed in the ingested conidia. In other words, the *Bb*-AaIT strain was capable of killing the larvae by ingesting the conidia in suspension and killing adult mosquitoes through both cuticle infection and conidia ingestion at a higher efficiency than the wild-type strain.

When fungal spores are applied to an aquatic habitat, which is typical for mosquito larvae, the nutrients in the water are usually sufficient for stimulating the germination of the spores following water intake (Bukhari et al. 2011; Hegedus and Khachatourians 1995). The scorpion toxin should be delivered into the insect circulatory system since it acts upon the injection of the scorpion venom into the prey or enemy (Matsumoto et al. 2014). For mosquito larvae, the primary path of infection was through the feeding, respiratory apparatus (Miranpuri and Khachatourians 1991) or cuticle. In this study, after the mosquito larvae were infected by *Bb*-AaIT, the scorpion toxin AaIT was delivered into the hemolymph by spore germination, which sped up the death of the mosquito larvae. When adult mosquitoes were exposed to *Bb*-AaIT by body contact, the fungal infection started from the adhesion of the conidia to the host cuticle, followed by germination and cuticle penetration into the hemocoel (Feng et al. 1994). Similarly, the expression of AaIT accelerated the death of adult mosquitoes. Moreover, when the adult mosquitoes were infected by feeding on 10% glucose solution containing *Bb*-AaIT conidia, the AaIT was expressed and released into the midgut environment by the ingested conidia, which accelerated the death of the infected adult mosquitoes (Matsumoto et al. 2014).

The effective use of fungi as bio-insecticides is largely dependent on the persistent activity of fungal spray residues, which can be influenced by some factors including the formulation, substrate, fungal isolate, UV radiation and the prevailing abiotic conditions (Acharya et al. 2015; Blanford 2012; Darbro and Thomas 2009; Jackson et al. 2010; Jaronski 2010; Jenkins and Thomas 1996; Morleydavies et al. 1996). A recent study stated that the persistence of *B. bassiana* products was reduced in the field compared with that observed in the laboratory; conidia remained viable for up to 3 months under laboratory conditions, but for only 1–2 weeks in the field (Acharya et al. 2015). An appropriate formulation is needed to ensure the even distribution of conidia throughout the treated area and to improve the persistent activity of conidia that were exposed to various environmental conditions. In this study, the transgenic strain (*Bb*-AaIT) was found to be significantly effective at controlling larval and adult mosquitoes. However, additional studies are required to find an appropriate formulation to exert the maximum potential entomopathogenic activity of *Bb*-AaIT against mosquitoes.

In conclusion, our study demonstrated that the virulence of wild type *B. bassiana* can be improved significantly by introducing the insect-specific toxin gene *AaIT* into the genome to express the exogenous toxin in the fungus. This recombinant *B. bassiana* was valuable for mosquito control, and even for other pest control.

## Additional file

**Additional file 1: Figure S1.** *AaIT* Gene synthesis and plasmid construction. **Figure S2.** Identification of genetic stability of *AaIT* gene in the different generations of recombinant *Beauveria bassiana.*

## Abbreviations

LC_50_: the median lethal concentration
WT: wild type strain
LT_50_: the median lethal time
CDA: Czapek’s agar
SDB: Sabouraud dextrose broth
GM: glucose-mineral
PPT: phosphinothricin
TCM: time–concentration–mortality
UV: ultraviolet
VGSCs: the voltage-gated sodium channels
