# Supplementary material for: Scorpion neurotoxin AaIT-expressing Beauveria bassiana enhances the virulence against Aedes albopictus mosquitoes
Source: AMB Express. 2017 Jun 9;7:121. doi: 10.1186/s13568-017-0422-1 (PMC5466577; doi:10.1186/s13568-017-0422-1)
Supplement: Supplementary file 1 — Additional file 1: Figure S1. AaIT Gene synthesis and plasmid construction. Figure S2. Identification of genetic stability of AaIT gene in the different generations of recombinant Beauveria bassiana. [file 13568_2017_422_MOESM1_ESM.docx]

**Additional file 1**

**
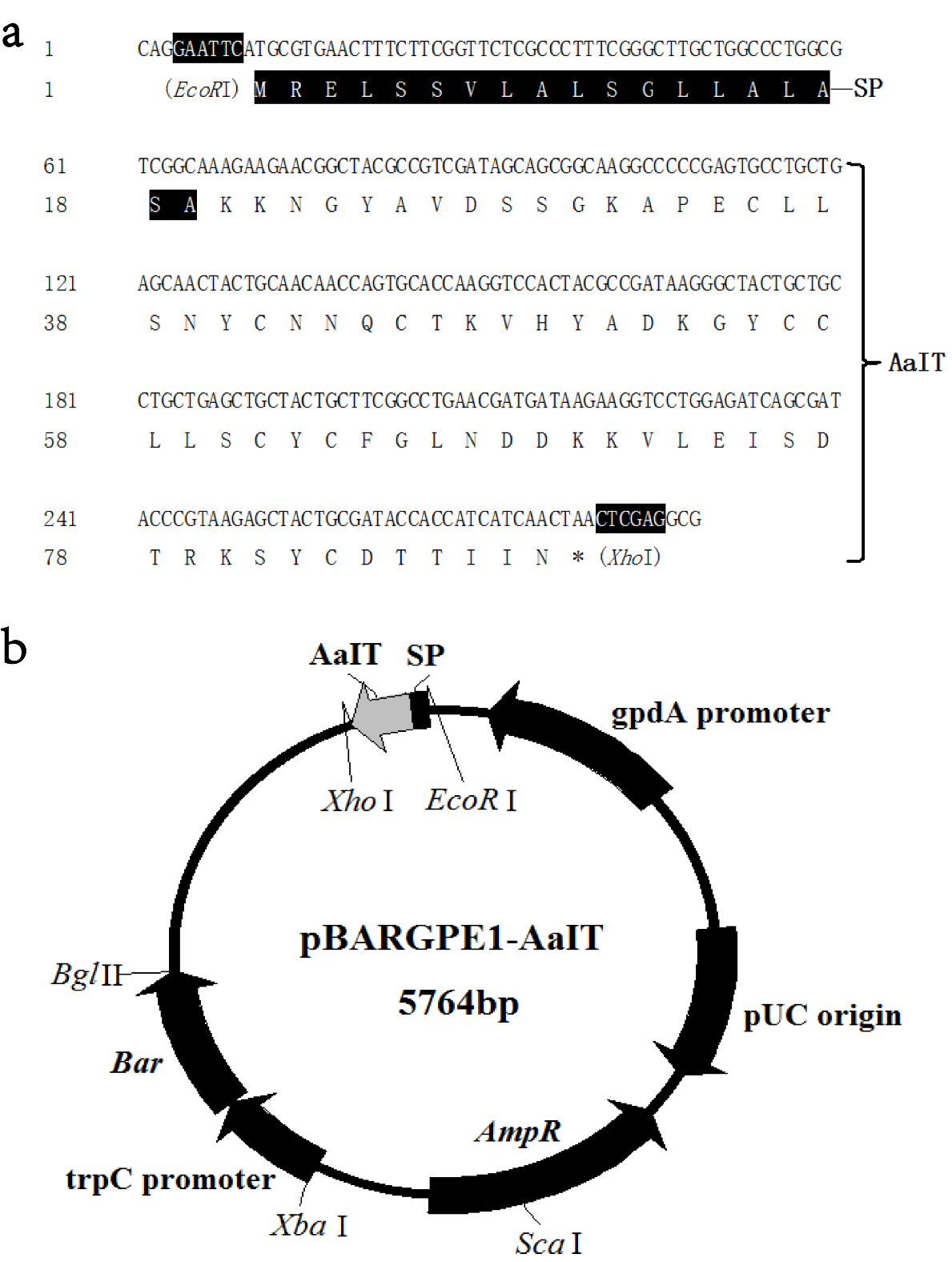
**

**Figure S1.** *AaIT* Gene synthesis and plasmid construction. (a) A codon-optimized synthetic sequence for the AaIT toxin gene fused with the signal peptide of [*Metarhizium anisopliae* Mcl1 promoter](http://www.baidu.com/link?url=IFPjCFERKGwGjOJVMB36-NC5t2o7By51ZEPeMVeB-7cOlnYTFtHdtWS6pFF4NiT170_VqMTfFdJ5RdF7FzJ7B_&wd=&eqid=fe3819b00002e74100000006586c5fde) (SP, shadowed). (b) A map of the pBARPE1-AaIT plasmid with the SP-AaIT insert. The plasmid retains the *BAR* and *AMP* resistance genes of the parent plasmid pBARGPE1.


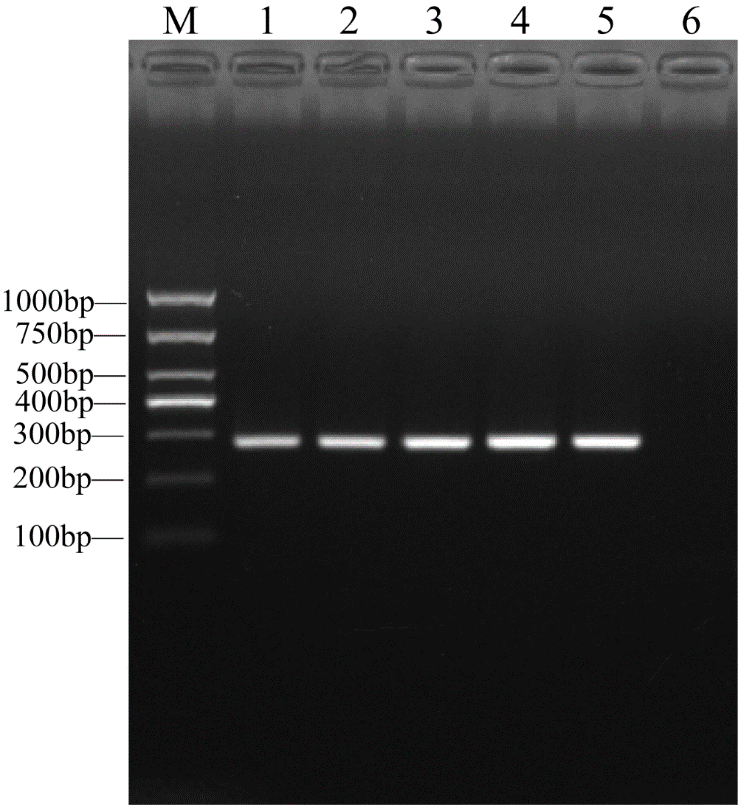


**Figure S2.** Identification of genetic stability of *AaIT* gene in the different generations of recombinant *Beauveria bassiana.*

Conventional PCR were performed with the genomic DNAs of the different generations of the recombinant *Bb*-AaIT and the WT. M, DNA maker; lanes 1-3, the transformants that were subcultured for 1 to 3 generations on CDA with 150 µg/mL PPT; lane 4, the transformants subcultured on CDA with 400 µg/mL PPT; lane 5, the positive control and lane 6, the negative control (WT).
